# Characteristics of Physical Exercise Programs and Their Effects on Quality of Life and Functional Capacity in Individuals with Chronic Obstructive Pulmonary Disease: A Scoping Review

**DOI:** 10.3390/medicina61060970

**Published:** 2025-05-23

**Authors:** Rafael Oliveira, João Paulo Brito, Halil İbrahim Ceylan, Maria de Brito Soares, Alexandre Duarte Martins, Tiago Vasconcelos, João Moutão, Susana Alves

**Affiliations:** 1 Santarém Polytechnic University, School of Sport, Av. Dr. Mário Soares, 2040-413 Rio Maior, Portugal; jbrito@esdrm.ipsantarem.pt (J.P.B.); af_martins17@hotmail.com (A.D.M.); 210500270@esdrm.ipsantarem.pt (T.V.); jmoutao@esdrm.ipsantarem.pt (J.M.); salves@esdrm.ipsantarem.pt (S.A.); 2 Research Center in Sport Sciences, Health Sciences and Human Development (CIDESD), Santarém Polytechnic University, Av. Dr. Mário Soares, 2040-413 Rio Maior, Portugal; 3 Physical Education and Sports Teaching Department, Faculty of Sports Sciences, Ataturk University, 25240 Erzurum, Turkey; halil.ibrahimceylan60@gmail.com; 4 Comprehensive Health Research Centre (CHRC), Departamento de Desporto e Saúde, Escola de Saúde e Desenvolvimento Humano, Universidade de Évora, Largo dos Colegiais, 7000-671 Évora, Portugal; 5 Department of Sports Sciences, University of Beira Interior, 6201-001 Covilhã, Portugal; 6 Sport Physical Activity and Health Research Innovation and Technology Center (SPRINT), Santarém Polytechnic University, Av. Dr. Mário Soares, 2040-413 Rio Maior, Portugal

**Keywords:** COPD, exercise training, cardiorespiratory fitness, strength training

## Abstract

*Background and Objectives:* Individuals with chronic obstructive pulmonary disease (COPD) often exhibit some degree of intolerance to physical exercise and several limitations in daily activities. Therefore, the objective of this study was to conduct a scoping review on the characteristics—frequency, intensity, time, and type (FITT)—and the effects of exercise programs on quality of life and functional capacity in individuals with COPD. *Materials and Methods:* The present review included 21 studies that were scoping-reviewed to describe their main findings and training characteristics. *Results:* The participants across studies ranged in age from ~39 to 76 years with mild to very severe COPD stages. The results showed that, among all studies, eleven used cardiorespiratory training (e.g., walking or cycling), five used strength training (e.g., exercises with elastic bands or traditional resistance training), and five implemented combined training (i.e., cardiorespiratory and strength exercises). *Conclusions:* Overall, all training protocols improved aerobic capacity (cardiorespiratory training), strength (resistance training), and both capacities together (combined training). In conclusion, this review provided complementary insights to existing exercise prescription guidelines, particularly concerning cardiorespiratory, strength, and combined training in individuals with COPD. However, the methodologies of the training protocols varied widely, and detailed descriptions of FITT components were often incomplete or lacking clarity, especially regarding the specific exercises used. Future research should include more comprehensive spirometry variables such as forced expiratory volume 1 or forced vital capacity, as these are critical for determining COPD stages. Thus, there is a clear need for more high-quality research with robust methodological design in the context of exercise interventions for individuals with COPD.

## 1. Introduction

Chronic obstructive pulmonary disease (COPD) is a persistent condition that blocks airflow in the lungs, is irreversible, underdiagnosed, and in some cases (early) fatal, and interferes with normal breathing capacity [1]. It evolves in degrees of severity (0 to 4) [2], and it is presented as the third leading cause of death worldwide [1,2].

COPD is associated with diseases such as asthma, cystic fibrosis, and interstitial lung disease. In addition, COPD is defined as a chronic inflammation of the small airways [3]. Asthma is characterized by a reversible obstruction to airflow and bronchial inflammation [4], while cystic fibrosis primarily affects the lungs, leading to chronic and recurrent infections [5].

The most significant consequence of COPD is a low oxygen level, which causes a narrowing of the intima of blood vessels (stenosis) that prevents blood from the right side of the heart from reaching the lungs, leading to an increase in pressure, known as pulmonary hypertension. Individuals with COPD are subject to develop heart rhythm abnormalities (arrhythmias), lung cancer, osteoporosis, depression, coronary artery disease, muscle wasting (atrophy), and anxiety [6]. Some signs of this condition are coughing, increased sputum purulence, fever, and nasal congestion [5].

The practice of physical exercise has been referred to as a complementary therapy to treat pulmonary diseases. For instance, pulmonary rehabilitation promotes improvements in functionality and exercise tolerance, thereby enhancing the quality of life for the individuals. Pulmonary rehabilitation should incorporate resistance exercises to enhance muscle strength, alleviate disease symptoms, improve functional capacity, and increase resistance to fatigue [7]. Corroborating the positive effects of pulmonary rehabilitation, some systematic reviews reported large and significant improvements in the clinical status of individuals, including increased tolerance to physical exercise, enhanced strength, and resistance to fatigue [8,9,10,11,12]. On the one hand, Lacasse et al. [8] analyzed the effects of rehabilitation on quality-of-life markers and exercise capacity related to walking tests. In this review, rehabilitation was considered as exercise training with a minimum duration of four weeks. Among the 31 randomized controlled trials included for analysis, these authors found significant improvements in the previous capabilities. It is worth noting that another systematic review analyzed several exercise tests, including the 6 min walk test, the incremental shuttle walk test, and the endurance shuttle walk test, in adults with chronic respiratory disease. This review confirmed the robustness of all tests in assessing functional exercise capacity in adults with COPD [13]. On the other hand, Iepson et al. analyzed the efficiency of combining resistance training and cardiorespiratory training compared with cardiorespiratory training alone, and the main findings were that both types of intervention were equally effective at improving quality-of-life markers and exercise capacity (using maximal oxygen uptake and exercise performance in watts) in individuals with COPD. However, it was also shown that additional resistance training contributed to higher improvements in leg muscle strength [9]. Furthermore, a recent systematic review compared the effectiveness of high- and low-moderate-load lower limb resistance training on muscle strength and exercise capacity. The authors showed that both types of intervention also improved muscle strength and might increase exercise capacity [10]. Older systematic reviews also demonstrated a positive effect on muscle function, although no specific guidelines regarding training characteristics were provided [11,12].

Regarding exercise training programs, the recommendations of the British Thoracic Society Pulmonary Rehabilitation Guideline Development Group and the British Thoracic Society Standards of Care Committee [14] for individuals with COPD suggested incorporating exercise training programs to complement pulmonary rehabilitation. They should consist of cardiorespiratory exercise, utilizing interval and continuous training methods, to enhance the individuals’ cardiorespiratory capacity and, consequently, improve lung function, thereby contributing to symptom reduction and an improvement in quality of life. In this regard, the American College of Sports Medicine [15] recommended engaging in at least 3–5 days of aerobic exercise, 2–3 days of resistance training, and 2–3 days of flexibility training per week. Aerobic exercise should be performed at a moderate to vigorous intensity (50–80% of peak work rate or 4–6 arbitrary units on the Borg CR10 scale). Resistance training should be performed at 60–70% of one repetition maximum (1RM) for beginners and at 80% of 1RM or higher for experienced trainers, while stretching should be performed to the point of feeling tightness or slight discomfort [15].

Despite the guidelines for exercise also providing details about duration with different intensities for aerobic exercise or range of sets and repetition for resistance and stretching exercises, such exercise recommendations were general and did not provide details about the exercise prescription and planning, which should include details about all specific exercises along with their intensity, duration (effort and rest periods), and training frequency [15]. Moreover, there are particular exercise prescription considerations to follow regarding the severity of COPD. For instance, individuals with mild COPD should engage in exercise according to the intensity guidelines recommended for healthy older adults. At the same time, those with moderate to severe COPD should apply intensities of greater than 60% of their peak work rate. Finally, individuals with severe COPD should engage in light-intensity aerobic exercise [15]. Considering the previous systematic reviews, it would be expected to find some suggestions for exercise training program recommendations. However, this is not observed [8,9,10,11,12]. Existing studies on exercise in COPD often report general outcomes (e.g., improved dyspnea or exercise capacity) but lack standardized reporting of FITT principles. A comprehensive synthesis of how each FITT component is used and how they relate to clinical outcomes (e.g., FEV1, quality of life, hospital readmission) is rarely provided. Moreover, they focused on quality of life, cardiorespiratory capacity, and muscle function, while other relevant outcomes for COPD, such as forced expiratory volume 1 (FEV1) and forced vital capacity (FVC), were not considered. This makes it hard for clinicians to translate findings into practice without a clearer map of what works best for whom. A better understanding of FITT in COPD rehab could lead to (i) improved guideline development; (ii) more tailored exercise prescriptions; (iii) greater clarity for future clinical trials on what parameters to report and optimize.

Therefore, the present study aims to fill the previous gaps by conducting a scoping review on the exercise training programs’ FITT (frequency, intensity, time, and type) characteristics and their effects in individuals with COPD. The goal is to map and categorize existing evidence, not necessarily to assess the effectiveness of one intervention over another. The present study allows for identifying research gaps, clarifying definitions, and summarizing what is known and unknown about the application of different FITT characteristics in COPD.

Based on the benefits of exercise training programs in individuals with COPD, as well as the challenges they present, this study aims to describe the details of prescribing and planning these various programs, as well as their effects on quality of life.

## 2. Materials and Methods

### 2.1. Design

The present study is a scoping review, conducted in accordance with the items of the Preferred Reporting Items for Systematic Reviews and Meta-Analyses 2020 (PRISMA 2020) [16] guidelines for performing systematic reviews in the sports sciences [17] and the PRISMA-ScR extension for scoping reviews [18]. The PRISMA 2020 checklist is included in File S1. The scoping review protocol was a priori registered in the OSF platform with the project number osf.io/vq2az and protocol registration DOI https://doi.org/10.17605/OSF.IO/BRQHD.

### 2.2. Eligibility Criteria

The following inclusion criteria met a PICOS strategy: (i) “P” (patient) corresponded to participants, aged ≥18 years, regardless of gender and ethnicity with COPD; (ii) “I” (intervention) corresponded to a physical exercise program performed in any person with COPD, with a minimum of six weeks of intervention; (iii) “C” (comparator) corresponded to the comparison between the control group (CG) versus the intervention group (IG); (iv) “O” (outcome) corresponded to the exercise plan, periodization, frequency, intensity, duration and type of exercise, as well as the benefits of the specific programs related to quality of life (e.g., physiological, biological) and/or functional capacity (e.g., functional tests such as 30 s chair stand); (v) “S” (study design) corresponds to randomized clinical trials (controlled clinical trial, CT, and randomized controlled trial, RCT). Only studies written in English were included.

The following exclusion criteria were applied: (i) samples with more than COPD pathologies or with individuals under 18 years of age; (ii) studies that encompassed other types of treatments in addition to physical exercise programs (e.g., pharmacological therapies, educational programs); (iii) studies that did not have a control group; (iv) effects of pharmaceutical treatments; (v) studies other than randomized clinical trials; (vi) studies that were not written in English.

### 2.3. Information Sources and Search Strategy

A systematic search of four databases (PubMed, Scopus, SPORTDiscus, and Web of Science [core collection]) was performed using the following keywords (Table 1):

(TI = ((exercise prescription*OR training program*OR exercise periodization*) AND (chronic obstructive pulmonary disease OR COPD*))).

These searches encompassed relevant publications available up to 25 November 2024.

### 2.4. Selection Process

The search for studies was conducted by two authors, H.I.C. and A.D.M. Then, screening and eligibility of the studies were conducted by two authors (H.I.C. and R.O.). In case of doubts, a third author (A.D.M.) was consulted. If studies did not meet the inclusion criteria, they were excluded. Abstracts with insufficient information regarding the inclusion and exclusion criteria were selected for full-text evaluation. In a second phase, two authors (H.I.C. and R.O.) independently evaluated all selected full-text articles and conducted a second round of selection, applying the inclusion and exclusion criteria. If any disagreement appeared, a third author was consulted (A.D.M.).

### 2.5. Data Collection Process

R.O. and T.V. extracted the data, while J.P.B. reviewed the process by cross-verifying the data to ensure accuracy and reliability. Data were systematically extracted according to a predefined template. If data were not available, the original authors were contacted to access the data.

### 2.6. Data Items

Population: name of first author, year of publication, number of participants, sex/gender, age, study type; intervention or exposure: study length in weeks.

Comparator: information about passive control groups or active control groups (namely, type of exercise, intensity, frequency, and duration).

Outcomes: the quality of life outcomes included (but were not restricted to) physiological, biological variables (e.g., cardiorespiratory-related measures such as maximal oxygen uptake, maximal heart rate and any pulmonary variables), neuromuscular-related measures such as muscular power and strength, balance and mobility-related measures, body characteristics and body composition (e.g., body mass index, lean mass, and fat mass), blood pressure (e.g., systolic and diastolic pressure), echocardiographic measures (e.g., cardiac output), bone health (e.g., bone mineral content), biochemical parameters (e.g., total cholesterol and glucose tolerance), and inflammatory parameters (e.g., leptin), and/or functional capacity (e.g., functional tests such as 30 s chair stand).

### 2.7. Study Risk of Bias Assessment

Despite the study risk of bias assessment not being applicable for scoping reviews [18], the Downs and Black tool, a validated instrument for assessing the methodological quality of healthcare interventions, was used to determine the validity and quality of individual studies [19]. This tool comprises 27 items, and item 27 has six possible scores. However, following Marquet et al. [20], this item was simplified. Specifically, if this item received a score of one, it meant that sufficient statistical power was achieved, while a score of zero meant that adequate power was not achieved. The remaining items were presented in the same manner, which means that a score of one indicated higher quality, and a score of zero indicated lower quality. Finally, if studies received a score of one on at least 50% of the items, then they were considered to be of sufficient quality and were included in the review [21]. The criteria for assessing study quality and bias are described in File S2.

## 3. Results

### 3.1. Selection of Sources of Evidence

**Figure 1:**
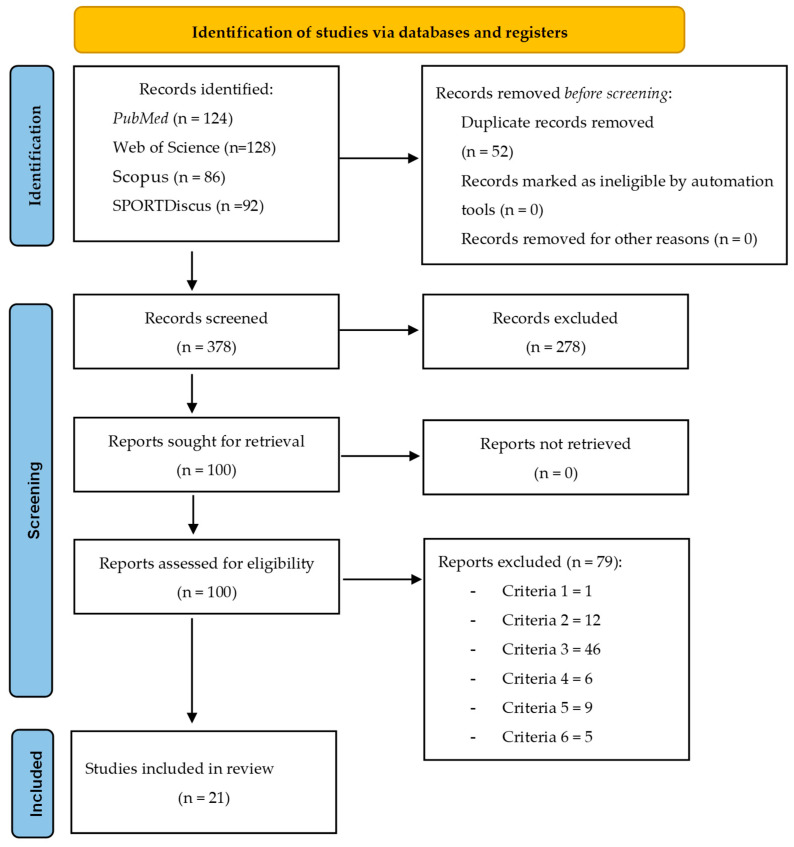
Flow diagram of the study.

A total of 430 articles were found across the four databases. All studies were exported using reference management software (EndNoteTM 20.0.1, Clarivate Analytics, Philadelphia, PA, USA). A total of 52 duplicate articles were recorded and subsequently removed. The remaining 378 articles were analyzed by their titles and abstracts. When insufficient information was available, the article was read in full, resulting in the removal of 278 articles deemed not to be within the scope of this review. Finally, after a thorough review of all articles, 77 additional articles were excluded because they did not meet the eligibility criteria. Thus, 21 articles were included in this scoping review (Figure 1).

### 3.2. Characteristics of Sources of Evidence

The studies covered an adult population diagnosed with COPD, with ages ranging from ~39 to 76 years (Table 2). In addition, studies included different types of COPD stages:

- mild [22];
- mild to moderate [23,24,25,26,27,28];
- mild to severe [29,30];
- moderate [31];
- moderate to severe [32,33,34,35,36,37,38,39];
- moderate to very severe [40];
- severe [41];
- severe to very severe [42].

Moreover, several instruments and tests were used to assess pulmonary function, body composition, aerobic capacity, strength, dyspnea, emotion, mastery, fatigue, and heart rate.

### 3.3. Risk of Bias Across Studies

Considering previous criteria [21], all studies received a score of one on at least 50% (final score ≥ 13.5), and for that reason, all studies were deemed sufficient in quality and were included in the review. The final score was a mean of 20.6 points (Table 3).

### 3.4. Intervention Characteristics

The characteristics of the intervention exercise programs and the primary outcomes are presented in Table 4. Regarding the exercises included, only the main phases of each training have been reported in the table. Moreover, the activity of the control groups was described. Overall, there were different exercise interventions such as cardiorespiratory training [25,26,28,30,32,33,35,36,37,38,40], strength training [22,29,34,39,42], and combined training [23,27,31,39,41].

**Table 2:** Characteristics of the studies.

Study	Aims	Participants/Age/Sex	Measures or Tests of the Study
Amin et al. [31]	To test the feasibility of a community-based exercise program and its effect on individuals with moderate COPD.	N: 19 (CG: 10; EG: 9) Age: CG: 72.0 ± 10.1|EG: 66.8 ± 8.1 Sex (M/F): CG: 6/4|EG: 6/3	Endurance time, SGRQ, body mass, body fat, Baseline Dyspnea Index, and total weight lifted.
Averna et al. [23]	To analyze the effects of a moderate-intensity exercise program in individuals with mild to moderate COPD.	N: 76 (EG: 56; CG: 20) Age: EG: 69 ± 5|CG: 71 ± 5 Sex (M/F): EG: 29/27|CG: 12/8	Pulmonary function measures (FVC, FEV1, FEV1/FVC), systolic/diastolic blood pressure, heart rate, oxygen saturation, workload symptoms, and SGRQ.
Bertolino et al. [29]	To evaluate the effects of a home-based resistance exercise program with elastic tubing after supervised resistance training on peripheral muscle strength and quality of life in individuals with mild to severe COPD.	N: 16 (EG: 7; CG: 9) Age: 64.31 ± 8.14 Sex (M/F): 13/3	CRDQ (dyspnea, emotion, mastery, and fatigue), maximal voluntary isometric contraction of knee extension, knee flexion, shoulder flexion, and shoulder abduction.
Borghi-Silva et al. [32]	To evaluate the effects of a 6-week aerobic exercise training program on autonomic modulation of heart rate in individuals with moderate to severe COPD.	N: 40 (EG: 20; CG: 14) Age: 67 ± 10 Sex (M/F): EG: 13/7|CG: 12/8	Pulmonary function measures (tidal volume, respiratory rate, ventilation), VO_2_, heart rate, blood lactate, dyspnea (RPE, CR10 scale), 6MWT, time domain (rMSSD and rMSM), and frequency domain (LF, HF, and LF/HF).
Borghi-Silva et al. [33]	To analyze the effects of a physical training program on cardiac autonomic modulation by linear and nonlinear heart rate variability indices and exercise capacity in individuals with moderate to severe COPD	N: 20 (EG: 10; CG: 10) Age: EG: 67 ± 7|CG: 66 ± 10 Sex (M/F): EG: 7/3|CG: 5/5	Pulmonary function measures (ventilation, VC, and FVC), heart rate, 6MWT, VO_2_, and VCO_2_.
Brønstad et al. [24]	To evaluate high-intensity knee extensor training on muscle function in individuals with mild to moderate COPD.	N: 12 (EG: 7; CG: 5) Age: EG: 67.6 ± 7.2|CG: 70.0 ± 4.6 Sex (M/F): ND	Mitochondrial respiration of the vastus lateralis muscle, VO_2_peak, work economy, knee-extensor testing (peak work, quadriceps muscle mass, femoral blood flow at 6 watts L·min^−1^, femoral blood flow at peak work, muscle VO_2_ at 6 watts, muscle VO_2_ at peak work, pulmonary VO_2_ at 6 watts, pulmonary VO_2_ at peak work, lactate at 6 watts, and lactate at peak work).
Calik-Kutukcu et al. [34]	To evaluate the effects of arm strength training on arm exercise capacity, activities of daily living, and occupational performance in individuals with moderate to severe COPD.	N: 42 (EG: 21; CG: 21) Age: EG: 58.38 ± 9.32|CG: 59.71 ± 9.3 Sex (M/F): EG: 16/5|CG: 11/10	Hand grip strength, heart rate, SpO_2,_ DSpO_2_, dyspnea (CR10 scale), arm fatigue (CR10 scale), leg fatigue (CR-10 scale), general fatigue (CR10 scale), peak workload, VO_2_peak, blood pressure, and upper extremity disability (Milliken Activity Daily Living Scale).
Clark et al. [22]	To analyze the effects of strength training on muscle function, isokinetic skeletal muscle, and cardiorespiratory capacity in individuals with mild COPD.	N: 43 (EG: 26; CG: 17) Age: 49 ± 11 Sex (M/F): EG: 15/1|CG: 10/7	Pulmonary function measures (VEmax, VTmax, respiratory rate, FEV1, RV), isokinetic muscle strength of upper and lower limb using maximal contraction test, average peak torque and sustained repeated contractions, VO_2max_, heart rate maximum, endurance walk test, body mass index, PAO_2_, and Borg scale.
Costes et al. [25]	To evaluate the effects of an exercise training program on baroreflex sensitivity in individuals with mild to moderate COPD.	N: 39 (EG: 21; CG: 18) Age: 62 ± 9|66 ± 1 Sex: ND	Pulmonary function measures (FEV1, VC, FEV1/VC, RV, TLC, ventilation), PAO_2_, PACO_2_, workload, VO_2_peak, VCO_2_, heart rate, spontaneous breathing, and head-up tilt (total frequency power, low frequency, high frequency).
Frei et al. [42]	To assess the effectiveness of the program and the experience of participants after 12 months in individuals with severe and very severe COPD who completed pulmonary rehabilitation.	N: 123 (EG: 61; CG: 62) Age: EG: 66.1 ± 8.3|CG: 67.4 ± 7.9 Sex (M/F): EG: 30/31|CG: 32/30	Chronic Respiratory Questionnaire, 1 min sit-to-stand test, and 6MWT.
Gelinas et al. [26]	To analyze whether periodized aerobic exercise training could improve vascular structure and function in individuals with mild to moderate COPD.	N: 44 (EG: 24; CG:20) Age: EG: 69 ± 7|CG: 64 ± 5 Sex: ND	Pulmonary function measures (FEV1, FVC, FEV1/FVC ratio, TLC, RV, RV/TLC ratio, FRC, diffusion capacity for carbon monoxide, and diffusion capacity corrected for alveolar volume), endothelial function through brachial arterial flow-mediated dilatation, peripheral pulse wave velocity, carotid artery intima-media thickness, carotid compliance, distensibility, β-stiffness index, and VO_2_peak.
Gouzi et al. [27]	To compare changes in blood pressure during exercise in individuals with mild to moderate COPD, about muscle capillarization.	N: 84 (EG: 49; CG:35) Age: EG: 61.5 ± 7.9|CG: 62.1 ± 5.8 Sex (M/F): EG: 30/19|CG: 18/17	Pulmonary function measure (FEV1), body mass index, 6MWT, VO_2SL_, resting and maximum systolic/diastolic blood pressure, muscle biopsies, capillary density, and capillary-to-fiber ratio.
Ko et al. [41]	Whether a short course of exercise training post-AECOPD, with periodic reinforcement exercise training and phone call reminders, reduces readmissions and increases physical activity in severe COPD individuals.	N: 136 (EG: 68; CG 68) Age: EG: 76 ± 8|CG: 74 ± 7 Sex (M/F): EG: 67/1|CG: 65/3	The average number of steps per day, MET, average daily wearing time in minutes, percentage of daily wear time, percentage of time spent in sedentary intensity, light intensity, moderate intensity, and vigorous to very vigorous intensity. Pulmonary function measures (FEV1, FVC), body mass index, 6MWT, COPD assessment test, modified Medical Research Council score, and SGRQ.
Leite et al. [30]	To analyze the effects of 12-week aerobic training using continuous and interval sessions on autonomic modulation, mucociliary clearance, and aerobic function in individuals with severe, moderate, and mild COPD.	N: 16 (EG: 10; CG:6) Age: EG: 62|CG: 62.5 Sex: ND	Pulmonary function measures (FEV1, FVC, and FEV1/FVC ratio), heart rate variability measures (standard deviation of regular RR intervals, root mean square of the difference between the adjacent regular RR intervals in a time interval, low frequency power, low frequency power in normalized units, high frequency power, high frequency power in normalized units, LF/HF ratio), SpO_2_, subjective perception of effort, VO_2_peak, and gas exchange threshold.
Liu et al. [35]	To analyze the effects of a supervised endurance exercise training program in a home setting, offering convenience and prolonged benefits, in individuals with moderate to severe COPD.	N: 48 (EG: 24; CG: 24) Age: EG: 71.4 ± 1.7|CG: 72.8 ± 1.3 Sex: M	Pulmonary function measures (FVC, FEV1, FEV1/FVC, inspiratory capacity), incremental shuttle walk test, breathlessness (Borg scale), Short-Form-12 quality-of-life questionnaire.
Marrara et al. [36]	To evaluate the responsiveness of the 6MST to an aerobic physical training program and to determine its efficacy regarding spirometry variables during the 6MST, as well as physical performance, sensation of dyspnea, and SpO_2_ during the 6MST and 6MWT, in individuals with moderate to severe COPD.	N: 36 (EG: 21; CG:15) Age: EG: 70.5 ± 8.5|CG: 68.3 ± 8.7 Sex: ND	Pulmonary function measures (FEV1, FVC, FEV1/FVC, maximal voluntary ventilation), 6MST, VO_2_peak, VO_2_, ventilatory equivalent for CO_2_ at rest, steps climbed, work efficiency, dyspnea (modified Borg scale), SpO_2_ levels, 6MWT, and dyspnea (modified Borg scale).
Pothirat et al. [40]	To determine the long-term efficacy of an intensive cycle ergometer exercise program on various clinical parameters of individuals with moderate, severe, or very severe COPD.	N: 41 (EG: 27; CG: 14) Age: EG: 71.9 ± 6.4|CG: 72.0 ± 7.9 Sex (M/F): EG: 12:15|CG: 7:7%	Pulmonary function measures (FEV1 and FEV1/FVC), as well as upper and lower limb muscle strength, were measured using a handheld dynamometer. Maximum inspiratory pressure, 6 min walk test (6MWT), Modified Medical Research Council scale, Transitional Dyspnea Index questionnaire, SGRQ, and survival rates were also assessed.
Varas et al. [28]	To assess the effects of a community-based pulmonary rehabilitation program designed to increase physical activity in participants with mild to moderate COPD.	N: 40 (EG: 21; CG: 19) Age: EG: 69.5 ± 7.4|CG: 64.8 ± 9.1 Sex (M): EG: 18/3|CG: 13/6	Endurance shuttle test, steps/day recorder device, modified Baecke questionnaire, and SGRQ.
Wang et al. [37]	To investigate whether a home-based exercise training program can reduce inflammatory biomarkers in individuals with moderate to severe COPD.	N: 26 (EG: 12; CG: 14) Age: EG: 71.4 ± 1.9|CG: 71.9 ± 2.7 Sex: M	Pulmonary function measures (FEV1 and FEV1/FVC ratio), shuttle walk test, elbow flexion and knee extension muscle strength through a dynamometer, serum C-reactive protein, and the concentration of interleukin-8.
Wootton et al. [38]	To evaluate the effect on health-related quality of life (HRQoL) of adding ongoing feedback to a 12-month unsupervised maintenance walking program in individuals with moderate to severe COPD.	N: 95 (EG: 49; CG: 46) Age: EG: 70|CG: 69 Sex (M/F): EG: 25/24|CG: 30/16	Pulmonary function measures (FEV1, FVC, FEV1/FVC, TLC, FRC, RV, and RV/TLC), carbon monoxide diffusion capacity, SGRQ, the Chronic Respiratory Disease Questionnaire, 6MWT, incremental shuttle walk test, and endurance shuttle walk test.
Zambom-Ferraresi et al. [39]	To compare the effects of 12-week training periods involving resistance training only with the impact of 12-week training periods involving combined resistance and endurance training on strength, endurance performance, and quality of life in individuals with moderate to severe COPD.	N: 36 (EG1: 14; EG2: 14; CG: 8) Age: EG1: 68 ± 7|EG2: 68 ± 7|CG: 69 ± 5 Sex: ND	Pulmonary function measures (FEV1, FVC, FEV1/FVC, TLC, IC, and IC/TLC), maximum inspiratory pressure in cm H_2_O (MIP), maximum expiratory pressure in cm H2O (MEP), maximal power output (50% of one repetition maximum in the leg press), maximal strength (in the leg-press and chest-press), maximal dynamic strength, maximal cycle exercise test, and the BODE index.

COPD, chronic obstructive pulmonary disease; CG, control group; EG, experimental group; F, female; M, male; SGRQ, St. George’s Respiratory Questionnaire; VC, vital capacity; FVC, forced vital capacity; FEV1, forced expiratory volume in the first second; TLC, total lung capacity; RV, residual volume; FRC, functional residual volume; VO_2_, oxygen uptake; rMSSD, square root of the mean of the sum of the squares of differences between adjacent normal R-R intervals; rMSM, standard deviation of R-R intervals; LF, low frequency in normalized units; HF, high frequency in normalized units; LF/HF: low frequency/high frequency ratio; VCO_2_, carbon dioxide output; TDI, transition dyspnea index; 6MWT, 6 min walking test; CRDQ, Chronic Respiratory Disease Questionnaire; SpO_2,_ oxygen saturation; DSpO_2,_ carbon dioxide saturation; VEmax, ventilation maximum; PAO_2_, arterial oxygen tension; PACO_2_, dioxide carbon tension; VO_2SL_, symptom-limited oxygen uptake; MET, metabolic equivalent task; 6MST, six-minute step test.

**Table 3:** Assessment of the risk of bias.

Reference	Reporting										Ext. Validity			Int. Validity							Int. Validity-Cofounding						Power	Final
	1	2	3	4	5	6	7	8	9	10	11	12	13	14	15	16	17	18	19	20	21	22	23	24	25	26	27	Score
Amin et al. [31]	1	1	1	1	1	1	1	1	1	1	1	1	0	0	1	1	1	1	1	0	1	1	1	0	1	1	1	22
Averna et al. [23]	1	1	1	1	0	1	0	0	1	1	1	1	1	0	0	1	1	1	1	1	1	1	1	0	0	1	0	19
Bertolini et al. [29]	1	1	1	1	1	1	0	1	0	1	1	1	1	0	0	0	1	1	1	1	1	1	0	0	0	1	0	18
Borghi-Silva et al. [32]	1	1	1	1	1	1	0	1	1	0	1	1	0	0	0	1	1	1	1	1	1	1	1	0	0	1	1	20
Borghi-Silva et al. [33]	1	1	1	1	1	1	0	1	1	0	1	1	0	0	0	1	1	1	1	1	1	1	1	1	0	1	1	21
Brønstad et al. [24]	1	1	1	1	1	1	0	0	0	1	1	1	1	0	0	1	1	1	1	1	0	1	0	0	0	1	1	18
Calik-Kutukcu et al. [34]	1	1	1	1	1	1	0	1	1	1	1	1	1	0	1	0	1	1	1	1	1	1	1	0	0	1	1	22
Clark et al. [22]	1	1	1	1	1	1	1	0	0	1	1	1	1	0	0	1	1	1	1	1	1	1	0	0	0	0	0	18
Costes et al. [25]	1	1	1	1	0	1	0	1	1	1	1	1	1	0	0	1	1	1	1	1	0	1	0	0	0	1	0	18
Frei et al. [42]	1	1	1	1	1	1	1	1	1	1	1	1	1	0	0	1	1	1	1	1	1	1	1	1	1	1	1	25
Gelinas et al. [26]	1	1	1	1	1	1	1	1	1	1	1	1	1	0	0	1	1	1	1	0	1	1	1	0	1	1	1	23
Gouzi et al. [27]	1	1	1	1	1	1	1	1	0	0	1	1	1	1	0	0	1	1	1	1	0	1	0	0	1	0	0	18
Ko et al. [41]	1	1	1	1	1	1	1	1	1	1	1	1	1	0	0	1	1	1	1	1	1	1	1	0	0	1	1	23
Leite et al. [30]	1	1	1	1	1	1	0	1	1	1	1	0	0	0	1	1	1	1	1	1	1	1	0	0	0	1	0	19
Liu et al. [35]	1	1	1	1	1	1	0	1	1	0	1	1	1	0	0	1	1	1	1	1	1	1	1	0	0	1	1	21
Marrara et al. [36]	1	1	1	1	1	1	1	0	1	0	1	1	1	0	0	0	1	1	1	1	1	1	1	0	0	1	0	19
Pothirat et al. [40]	1	1	1	1	1	1	0	0	1	0	1	1	1	1	0	1	1	1	1	1	1	1	1	0	0	1	1	21
Varas et al. [28]	1	1	1	1	1	1	1	1	1	1	1	1	1	1	1	1	1	1	1	1	1	1	1	0	1	1	0	25
Wang et al. [37]	1	1	1	1	1	1	0	1	1	1	1	1	0	0	0	1	1	1	1	1	1	1	1	0	0	1	0	20
Wootton et al. [38]	1	1	1	1	1	1	1	0	1	1	1	1	0	0	1	1	1	1	1	1	0	1	1	0	1	1	1	22
Zambom-Ferraresi et al. [39]	1	1	1	1	1	1	0	0	1	0	1	1	1	0	1	0	1	1	1	1	1	1	1	0	0	1	1	20

Note: A detailed explanation of the domains and categories, together with instructions for the analysis, can be found in the original article [19] and File S1.

**Table 4:** Characteristics of the exercise training protocols.

Study	Program Duration, Frequency, Session Duration	Training Program	Main Outcomes
Amin et al. [31]	Program duration: 12 weeks Frequency: 2× week Session duration: 30 min plus resistance training	EG training program: - 30 min of continuous aerobic (in the target heart rate range ND); - Resistance training (muscle groups of the upper and lower body—conventional free weight or machine-based; the goal was to maintain 12 reps at 70–75% of the 1RM weight); CG activity: continuation of daily living activities.	The community-based exercise program was both feasible and effective in improving aerobic capacity (endurance time) and reducing the transition dyspnea index.
Averna et al. [23]	Program duration: 12 weeks Frequency: 3× week Session duration: 60 min	EG training program: - combination of free weights exercises, with equipment and isotonic machines, performed as separate sessions and in the form of circuits (30 min); - strength exercise (3× week/10–30 mint); - 1–3 sets, 15–20 reps of both single and multi-joint exercises (50% of 1RM); - Rest periods: 1st month (time needed to return to HR > 20–30% the rest HR; 2nd month ranged from 60 to 90 s; - Aerobic activity (20–45 min) at the intensity of 40–50% of HRR. CG training program: no physical training.	Favorable modifications in respiratory and cardiovascular parameters were observed, including improved exercise tolerance, reduced blood pressure values at rest and during exertion, increased forced vital capacity, higher oxygen saturation levels during exercise, and enhanced quality of life related to overall health.
Bertolini et al. [29]	Program duration: 16 weeks Frequency: 3× week Session duration: ND	EG training program (unsupervised): - The routine was controlled through the phone. - Training prescription (initial 4 weeks): 2 sets, progressively increased by adding one set every two sessions (maximum of seven sets), after which a new prescription was implemented. - Repetition number and load were determined based on a resistance fatigue test. - Material: elastic tubing (Lemgruber^®^ brand, Brazil). - Exercises: knee flexion, knee extension, shoulder flexion, and shoulder abduction. CG activity: instructed not to perform any physical activity.	The exercise training program maintained the gains made in the previous supervised training on the strength of the upper and lower limbs and CRDQ domains, with no additional benefits.
Borghi-Silva et al. [32]	Program duration: 6 weeks Frequency: 3× week Session duration: 30 min.	EG training program: - 30 min—treadmill ambulation (70% of the maximal speed). CG training program: no physical training.	Significant improvements in VO_2_peak, blood lactate, minute ventilation, dyspnea at peak exercise, sympathetic activity, and parasympathetic activity at rest. Moreover, reductions in respiratory rate and increases in tidal volume were observed during exercise.
Borghi-Silva et al. [33]	Program duration: 6 versus 12 weeks Frequency: 3× week Session duration: 35 min	EG training program: High-intensity workload relative to each subject’s aerobic capacity (walking). - Initially at an intensity of 70% (30 min). - Intensity was increased 5 km/h when the patient scored less than 4 on the Borg 0–10 scale. - Measures were repeated at the 6th week. CG activity: only received respiratory therapy (inhalation, desobstructive technique, and coughing).	After 6 weeks, HRV indices, aerobic capacity (as measured by walking distance), peak heart rate, and lactate levels improved. However, after 12 weeks, the majority of the variables continued to show additional improvements. Short-term rehabilitation is an adequate time to modify outcomes, as cardiac modulation and exercise capacity are beneficial.
Brønstad et al. [24]	Program duration: 6 weeks Frequency: 3× week Session duration: ND	- High-intensity interval aerobic knee extensor exercise training. - Knee extensor peak work testing and exercise training. - Four intervals of 4 min at 90% of peak work rate. - Each interval was separated by two minutes of active unloaded kicking. - A kicking frequency of 60 kicks per minute was pursued. - Both legs were exercised separately. CG activity: did not participate in the exercise training.	High-intensity aerobic interval training of a limited muscle group restored work performance and oxidative capacity of the quadriceps muscle.
Calik-Kutukcu et al. [34]	Program duration: 8 weeks (23 sessions) Frequency: 3× week Session duration: ND	EG training program: - Horizontal shoulder adduction, arm ergometer, shoulder flexion and abduction, elbow extension, elbow flexion, scapular elevation (trapezius), shoulder hyperextension (latissimus dorsi). - 8–12 reps of free weights at 40–50% of 1RM (number of reps increased when participant could complete 12 reps with the same load in two consecutive sessions (>12). - Loads were increased by 250 or 500 g per week. - Rest intervals of 2–3 min were given between sets. - Warm-up before and cool-down after the training sessions. CG activity: only breathing exercises.	The upper extremity muscle strength training alone increased upper extremity muscle strength, arm exercise capacity, performance in activities of daily living, and participants’ satisfaction with their performance in these activities. Additionally, it decreased dyspnea and fatigue perceptions during supported arm exercises and dyspnea perception during activities of daily living.
Clark et al. [22]	Program duration: 12 weeks Frequency: 2× week Session duration: ND	EG training program: - 3 sets of 10 repetitions. - 8 exercises (load at 70% 1RM). - Bench press (triceps); body squat (quadriceps); squat calf (medial and lateral gastrocnemius; soleus); latissimus (latissimus dorsi); arm curls (biceps); leg press (quadriceps, hamstrings, gluteals); knee flexion (quadriceps); hamstrings. - After 6 weeks, new 70% maximum value was determined. CG activity: no exercise intervention.	There was a significant reduction in skeletal muscle endurance and strength compared with healthy but sedentary individuals. Moreover, skeletal muscle training resulted in a highly significant improvement in exercise endurance during treadmill walking, without any central cardiorespiratory changes, and correlated with improved skeletal muscle function measures.
Costes et al. [25]	Program duration: 8 weeks Frequency: 3× week Session duration: 35/45 min	Endurance exercise training program: - Cycling exercise (30 min) (1st week). - Workload 60% (intensity increased to reach 75% at the end of the 2nd week). - Final six weeks: intensity maintains heart rate constant, and session duration increased to 40 min. CG activity: no training intervention.	Exercise training was associated with an increase in spontaneous baroreflex sensitivity, indicating cardiovascular benefits. It increased tolerance and the ability to sustain a high ventilation level without changes in pulmonary volumes or blood gas levels.
Frei et al. [42]	Program duration: 12 months Frequency: 6× week Session duration: 20 min	EG training program: - Exercises: trunk, upper limb, lower limb with different intensity levels. - Supervised by a coach. CG activity: no intervention and usual care.	The home exercise program did not affect dyspnea but improved 1MSTST performance and patient-perceived fitness.
Gelinas et al. [26]	Program duration: 8 weeks (24 sessions) Frequency: 3× week Session duration: 20–45 min	EG training program: Supervised training on the cycle ergometer. - Initial intensity (50% peak workload). - Weeks 1–4 intensity (50–75% W_max_). Weeks 5–8: - Hard (80–95% W_max_ for 3 min; 3 min rest at 40–45% W_max_) (5–6 interval) - Moderate (50–80% W_max_ for 40–45 min) - Hard moderate (55–85% W_max_ for 35–45 min) CG activity: no training intervention	Eight weeks of aerobic training improved cardiorespiratory fitness, as measured by VO2peak, reduced dyspnea, and lowered blood pressure, but had a non-significant effect on other established markers of cardiovascular disease risk.
Gouzi et al. [27]	Program duration: 6 weeks Frequency: 3× week Session duration: 1 h–30 min	Endurance training: - 20 sessions of endurance exercise (cycling or walking); - 4–6 weeks; - 45 min; - 10 min intensity of the ventilatory threshold + 5 min of active recovery; - Strength-building exercise; - 30 min; - 8–10 exercises (ND); - 10 sets; - 15 reps; - Intensity at 40% of isotonic 1RM. CG activity: followed a similar training program.	Participants showed slight improvements in VO_2_peak.
Ko et al. [41]	Program duration: 4–8 weeks (continuing exercise at home for 1 year) Frequency: 1–2× weekly Session duration: 2 h	- Supervised and individualized physical training program: - 60–70% of HRmax; - Adjusted on tolerability (SpO_2_); - Treadmill walking/running; - Upper and lower limb weightlifting (ND); - Stretching exercises. CG activity: received neither physiotherapy training nor phone calls.	A short course of exercise training following acute exacerbations of COPD, combined with periodic reinforcement and phone call reminders, reduced the frequency of exacerbations and increased the time to readmission for acute exacerbations of COPD. However, the program did not improve physical activities and exercise tolerance at 12 months.
Leite et al. [30]	Program duration: 12 weeks Frequency: 3× week Session duration: ND	EG training program performed on the treadmill: - Zone 1: 50 min sessions at an intensity (60% of VO_2_peak) (continuous effort); - Zone 2: 30 min sessions at an intensity (75% of VO_2_peak) (continuous effort); - Zone 3: formed by five efforts of 3 min performed at 100% of VO_2_peak; - 1 min of passive recovery (interval effort). 1st mesocycle: - Zone 1—85.9% total sessions; 344 min; - Zone 2—14.1% of total sessions; 57 min. 2nd mesocycle: - Zone 1—34.5% of total sessions; 138 min; - Zone 2—65.5% of total volume; 262 min. 3rd mesocycle: - Zone 2—32.5% of total volume; 130 min; - Zone 3—67.5% of total volume; 270 min. CG activity: no training.	Aerobic training (continuous and interval sessions) positively influenced the high frequency index (ms^2^), VO_2_peak, and anaerobic threshold.
Liu et al. [35]	Program duration: 3 months Frequency: daily Session duration: ND	EG training program: - Walk at a speed controlled by the tempo of music. - Exercise training intensity was set at 80% of the maximal capacity of the shuttle walking test. - Participants were required to maintain their walking speed until they could no longer keep up. - Supervision via cell phone. CG activity: same protocol and telephone reinforcement every 2 weeks (1st 3 months).	Participants in the cell phone group improved their shuttle walking test distance and duration after eight weeks. The improvements in shuttle walking test distance, inspiratory capacity, and quality-of-life questionnaire scoring improved even more at the end of the 12 weeks of training, with fewer acute exacerbations and hospitalizations.
Marrara et al. [36]	Program duration: 6 weeks Frequency: 3× week Session duration: 35 min	EG training program: - Treadmill aerobic physical training (30 min); - Speed was 70% of the max speed during CPET; - Supervised physical therapist. CG activity: - Respiratory therapy; - Education on diaphragmatic breathing; - Free arm; - Leg exercises; - Stretching neck, trunk, arm, leg.	The 6 min step test improved physical performance and reduced the sensation of dyspnea.
Pothirat et al. [40]	Program duration: 24 months Frequency: 2× per week Session duration: 30–60 min	EG exercise program: - Upper and lower limb cycle ergometer exercise training. - First 2 weeks included arm and leg cycling, trained for 30–40 min (30–35% HRR). - Pushing to the limits of their dyspnea, without exceeding 6 RPE. - Exercise sessions were increased in duration and intensity by 5 min and 5% HRR, alternately, every 2 weeks until a maximum of 50–60 min duration and 50–55% HRR. - Supervised by physiotherapists and nurses. CG activity: self-exercise at home.	All individuals showed strength increases in the four trained muscle groups. There were also improvements in the level of dyspnea, endurance, and quality of life over the 12-month follow-up period.
Varas et al. [28]	Program duration: 8 weeks Frequency: 5× per week Session duration: 30–60 min	EG training program: - Walking 5× week; - 30–60 min (cycles of 15–20 min); - Speed based on the shuttle test; - Each week, increase the total number of steps (10–20%). CG activity: only recommendations to walk more every day.	There were significant improvements in the shuttle run test, number of steps, and Baecke scores of the physical activity questionnaire, while the St. George’s Respiratory Questionnaire scores decreased. These results remained evident after 3 and 12 months of follow-up.
Wang et al. [37]	Program duration: 6 months Frequency: daily Session duration: ND	EG training program: - Endurance walking under mobile phone guidance; - Speed at 80% max capacity in ISWT. CG activity: - Same exercise protocol; - Without any telephone reinforcement.	A mobile-phone-based system can provide an efficient home endurance exercise training program with improved exercise capacity, strength of limb muscles, and a decrease in serum C-reactive protein and interleukin-8.
Wootton et al. [38]	Program duration: 2 months Frequency: 3× per week Session duration: ND	EG and CG training program: - A 2-month supervised walking training program. - Only differed once they entered the 12-month maintenance phase. - The third group received usual medical care and did not participate in any exercise training. EG and CG training program (unsupervised): - 12 months of walking exercise; - Starting at the same duration achieved in the final week of supervised training; - Pace that elicited a dyspnea score of three to four on 0–10-point category-ratio dyspnea scale; - Telephone calls; biofeedback provided via a pedometer (EG only); - Progressive goal settings (EG only).	Following a 2-month supervised walking training program, ongoing feedback was no more effective than no feedback in maintaining quality of life during a 12-month unsupervised walking program.
Zambom-Ferraresi et al. [39]	Program duration: 12 weeks Frequency: 2× week Session duration: 90 min	Resistance training alone: Dynamic exercise: - Lower limb (leg press, knee extension, knee flexion); - Upper limb (chest press, seated row, shoulder press); - Loads (50–70% 1RM); - 6–12 reps; - 3–4 sets. Combined training: - 1× week of dynamic resistance exercise (the same resistance training); - Another day of endurance exercises; - Cycle ergometer and endurance cycling (20–35 min); - Intensity 40–85% W_max_; - 65–90% heart rate maximal. CG activity: instructed to continue their habitual physical activity as before.	The combined program produced greater improvements in muscle power output and exercise capacity than the resistance training program alone. Specifically, muscle power and maximal exercise capacity improved, while heart rate and blood lactate at a given submaximal workload decreased. Identical improvements were found in aerobic capacity for both training groups.

EG, experimental group; CG, control group; ND, non-described; rep, repetitions; 1RM, one repetition maximum; HR, heart rate; HRR, heart rate reserve; 6MWT, 6 min walking test; 1MSTST, 1 min sit-to-stand-test; W_max_, peak workload; SpO_2_, oxygen saturation; VO_2_peak, oxygen uptake peak; CPET, cardiopulmonary exercise testing; RPE, rating of perceived exertion; COPD, chronic obstructive pulmonary disease; ISWT, incremental shuttle walk test.

## 4. Discussion

This scoping review analyzed and described the characteristics of exercise training programs and their effects on individuals diagnosed with COPD. Regarding the main effects, findings generally showed that different exercise training programs improved respiratory, cardiorespiratory, and muscle strength parameters. However, such positive effects were not observed in all included studies. In this regard, it is also relevant to highlight that there were different exercise interventions with different characteristics, such as community-based exercise, aerobic exercise, high-intensity exercise, resistance exercise, combined training, and home-based training, which provide additional information compared to previous systematic review studies that only focused on cardiorespiratory, strength, or both interventions combined [8,9,10,11,12]. Nevertheless, due to the reasons above, a meta-analysis could not be conducted to avoid potential bias.

When considering the previous systematic reviews [8,9,10,11,12], the present scoping review provides additional information because the eligibility criteria were broader and more precisely defined, or because the previous ones were outdated [12]. For instance, one study defined respiratory rehabilitation as any training program with or without education and/or psychological support, and RCTs were not mandatory [8], whereas another study only compared combined training with cardiorespiratory training alone [9], which was deemed ineligible for the present scoping review. Topcuoğlu et al. only included studies with resistance training protocols for the lower limbs [10], while excluding studies with progressive resistance exercise, which would have included several other potential studies [11].

Nonetheless, the positive effects were discussed in relation to the different training protocol types and are presented in the following subsections: cardiorespiratory training [25,26,28,30,32,33,35,36,37,38,40]; strength training [22,29,34,39,42]; combined training [23,27,31,39,41]. Additionally, limitations, strengths, and suggestions for future research were also addressed.

### 4.1. Cardiorespiratory Training

As previously mentioned, the characteristics of the studies differed in the selected exercises, training frequency, duration, and intensity. For instance, Borghi-Silva et al. [32] applied only treadmill ambulation for 30 min at 70% of maximal speed, three times a week, for six weeks and observed several improvements in peak oxygen consumption, blood lactate, minute ventilation, dyspnea at peak exercise, sympathetic activity, and parasympathetic activity at rest. Moreover, reductions in respiratory rate and increases in tidal volume were observed during exercise. Similarly, another study used the same intensity on a treadmill for six weeks, with an additional 5 min of training, and the results showed identical findings in terms of aerobic capacity and a reduced sensation of dyspnea [36]. Another study also employed walking training with the same intensity, frequency, and duration of six to twelve weeks [33]. While it showed identical improvements in HRV indices and walking distance, peak heart rate, and lactate, these findings were even more pronounced after 12 weeks of training, which supports the notion that more extended periods of training can yield greater benefits. Other research also applied a training program on a treadmill over 12 weeks, three times a week, with varied intensities of 60–100% of VO2peak (see Table 3), and found positive effects on the HRV high-frequency index (ms²), VO_2_peak, and anaerobic threshold [30].

A home-based program that consisted of walking at 80% of maximal capacity (until participants could not continue) showed improvements in the shuttle walking test distance after 8 weeks, while after 12 weeks, it showed additional improvements in the same variable, as well as in the quality of life and inspiratory capacity [35]. Again, this study aligns with previous research [33], which found that a longer duration (in this case, 12 weeks) yields additional and more beneficial outcomes.

Another walking-based program [28] was performed for 8 weeks, five times a week, for 30–60 min, with an intensity based on a shuttle test [43], and found that there were significant improvements in shuttle run test, number of steps, and Baecke scores of the physical activity questionnaire, while the St. George’s Respiratory Questionnaire scores decreased. These results remained significant after 3 and 12 months of follow-up, although no control was performed on physical activity [28]. Nonetheless, such findings are positive and should be considered for exercise prescription. Similarly, Wootton et al. [38] applied another walking program (with intensity not specified) and found similar improvements in aerobic capacity and St. George’s Respiratory Questionnaire scores. The same study found that these results were sustained over the 12 months of follow-up (again, physical activity was not controlled). Finally, a 6-month walking program performed at 80% of maximal capacity with a daily frequency showed improvements in upper and lower limb strength, as well as a decrease in serum C-reactive protein and interleukin-8, which is associated with a reduction in systemic inflammation in COPD individuals [37] because interleukin-8 is an oxidant-sensitive protein and a proinflammatory cytokine [44]. C-reactive protein is a sensitive, acute-phase reactant that can be activated during systemic inflammation, and it is associated with muscle mass, strength, physical function, and disability [45]. This was the only study to analyze such markers. Even so, it reinforced the training mode of walking [37].

In addition, other studies employed different training modes, such as cycling ergometers [24,25,26,40]. Costes et al. [25] progressively increased the intensity (60–75% of maximal capacity) and duration (30–40 min) over the eight training weeks and found improvements in baroreflex sensitivity and peak oxygen consumption. However, no changes were observed in pulmonary function tests. Gelinas et al. [26] applied the same training frequency, but they varied the progression in terms of intensity and duration (see Table 4). The authors found that eight weeks of training improved oxygen consumption and reduced dyspnea, while also decreasing blood pressure values, which highlights the importance of training progression. A contrasting study, in terms of training frequency (twice a week), applied upper and lower limb cycle ergometer exercise training, with intensity varying between 30 and 55% of heart rate reserve and a duration of 30–60 min [40]. This study also analyzed different variables and noted that upper and lower strength increased, as well as improvements in dyspnea, endurance, and quality of life, which were more evident at 12 months. Such findings lead to the speculation that the maintenance of the program did not contribute to additional findings over time, as the main effects were noted at 24 weeks rather than at 12 months of training [40], which again supports the importance of progression, as mentioned in previous research [26]. The last approach using a cycle ergometer was performed by Brønstad et al. [24], who applied high-intensity interval training of a knee extensor exercise (four intervals of four minutes at 90% of peak work rate) three times a week for six weeks and found that this program was highly effective in restoring skeletal quadriceps function.

It is relevant to highlight that all cardiorespiratory training programs were conducted in older individuals [25,26,28,30,32,33,35,36,37,38,40]. For this reason, the findings and exercise prescriptions should be cautiously interpreted for older individuals with COPD.

### 4.2. Strength Training

Regarding strength training protocols, only five studies were found to apply this type of training alone [22,29,34,39,42]. Bertolini et al. [29] employed four exercises for the upper and lower limbs using elastic bands to assess the effects following a previous supervised strength training protocol [46], which demonstrated the maintenance of strength gains, as well as a non-significant impact on the Chronic Respiratory Disease Questionnaire. Still, this study showed that even with a lower intensity than recommended [15], several benefits were found. In contrast, Calik-Kutukcu et al. [34] employed a more traditional training program consisting of eight exercises performed at 40–50% of 1RM, with eight to twelve repetitions, three times a week, over eight weeks. While the improvements in strength were expected, the study also found that participants’ satisfaction increased with their performance and decreased in perceptions of dyspnea and fatigue during supported arm exercises, as well as in dyspnea perception during activities of daily living. The relevant findings of this study were that resistance training was applied exclusively to the upper extremity muscles [34]. This study reinforces the previous suggestion that it is possible to obtain benefits beyond strength by using lower intensities than those typically recommended [15]. Still, the differences in training prescription could be related to differences in the ages of participants, as Calik-Kutukcu et al. [34] analyzed individuals aged ~58–59 years, while Bertolino et al. [29] analyzed older individuals aged ~64 years. In comparison, Clark et al. [22] employed a different approach, increasing the number of analyzed weeks to 12 while reducing the training frequency to twice a week for adults (mean age, 49 years). In addition, the authors applied three sets of 10 repetitions in eight exercises performed at 70% of 1RM and found improvements in strength, oxygen consumption, and maximal heart rate [22]. Similarly, Zambom-Ferraresi et al. [39] applied resistance training for 12 weeks, twice a week, involving six exercises for both upper and lower limbs, with an intensity of 50–70%, and found improvements in muscle strength and aerobic capacity in older adults. These findings suggest that traditional resistance exercise can be applied, following ACSM guidelines. The remaining study opted for a longer longitudinal approach of 12 months for older individuals conducted at home [42]. Still, this study found improvements in training frequency to six times, with a duration of 20 min, using the same type of exercises. However, no specific information was provided about the other training characteristics. Even so, the study only found a positive effect in the 1 min sit-to-stand test, suggesting that progression, longer duration, and supervision may be key factors in achieving more substantial benefits.

### 4.3. Combined Training

Five studies employed a combined training program incorporating cardiorespiratory and strength training characteristics for older adults. [23,27,31,39,41]. Due to the significant differences in the training protocols, it is recommended to consult Table 4 for further clarification. Nevertheless, the general characteristics included continuous aerobic exercise (~30 min, 40–50% of heart rate reserve or 60–90% of maximal heart rate) and resistance training for upper and lower limbs, performed at 40–75% of 1RM, with six to twenty repetitions and one to four sets. Considering the main effects, all studies reported positive outcomes in at least some of their measured variables. For instance, Amin et al. [31] demonstrated improvements in aerobic capacity and dyspnea with a training frequency of just two sessions conducted over 12 weeks. Similarly, Zambom-Ferraresi et al. [39] applied a combined training program for 12 weeks, twice a week, and found improvements in muscle power and maximal exercise capacity, while also observing decreases in heart rate and blood lactate at submaximal workloads. Although with only six training weeks and a training frequency of three times, Gouzi et al. [27] reported only modest improvements in maximal oxygen uptake. Additionally, Averna et al. [23] employed a training frequency of three times per week. They observed improvements in blood pressure values at rest and during exertion, increased exercise tolerance, enhanced forced vital capacity, improved oxygen saturation during exercise, and a better quality of life, as measured by the St. George’s Respiratory Questionnaire. In comparison to Amin et al. [31], the findings of Averna et al. [23] suggest that lower intensity in both cardiorespiratory and strength training, combined with a training frequency of three times per week, yielded greater benefits in COPD-related variables, particularly those associated with respiratory function. In contrast to previous studies, Ko et al. [41] implemented a training program over four to eight weeks, with a frequency of one to two sessions per week and a session duration of 2 h. Their results showed a reduction in the frequency of exacerbations, although the time spent on hospital readmissions for acute exacerbations increased.

### 4.4. Limitations, Strengths, and Suggestions for Future Research

This scoping review presented some limitations: (1) there was a great disparity in training methodologies, involving different types of exercise training protocols concerning the FITT characteristics; (2) descriptions of exercise training programs were not always present or were not objective in terms of how they were structured, especially in the description of the exercises used; (3) there were different stages of COPD included in the studies (mild to very severe). Consequently, the previous limitations avoided the development of a meta-analysis.

Nonetheless, the present scoping review offers practical implications for clinical, fitness, and academic practices, providing additional information about the effects of physical exercise training programs while presenting detailed information on the FITT principles for exercise prescription. Finally, it also describes several tests and exercises that can be replicated by fitness or exercise physiologists.

Specifically, it was shown that walking training was the only type to improve walking distance, maximal oxygen uptake, and tidal volume and to decrease heart rate measures at rest or in submaximal intensities, dyspnea, respiratory rate during exercise, and St. George’s Respiratory Questionnaire scores. To achieve these benefits, it was recommended to walk three times a week, for at least 30 min, with an intensity between 60% and 100% of VO_2_peak, for a minimum of six weeks. However, better results were found with 12 weeks to six months of training, during which serum C-reactive protein and interleukin-8 levels decrease, indicating a reduction in systemic inflammation. Moreover, cycling was another valid type of exercise, where the main findings were evident after eight weeks of training, when performed three times a week at a minimum intensity of 30% of the heart rate reserve. The main findings consisted of improving maximal oxygen uptake, resistance in both upper and lower limbs, dyspnea, and quality of life, while decreasing blood pressure values. Better benefits were found with six months of training, while durations of twelve months did not find additional benefits.

Additionally, it was found that strength could be improved through different types of protocols, including those with lower intensities than usually recommended (e.g., elastic bands with no additional percentage of 1RM). In general, traditional protocols involve using 40–70% of 1RM for eight to twelve repetitions, two to three times a week, for a minimum of eight weeks, with exercises targeting both upper and lower limbs (four to eight exercises). Overall, this type of training contributed to better participant satisfaction with their performance, while decreasing perceptions of dyspnea and fatigue.

Finally, the general characteristics of combined training included continuous aerobic exercise (~30 min, 40–50% of heart rate reserve or 60–90% of heart rate maximal) and resistance training for upper and lower limbs performed at 40–75% of 1RM, six to twenty repetitions, one to four sets. This type of training protocol demonstrated improvements in maximal oxygen uptake, dyspnea, exercise tolerance, forced vital capacity, oxygen saturation, and quality of life, while also reducing resting heart rate and blood pressure.

As suggestions for future research, it is recommended that studies assess additional spirometry variables, such as forced expiratory volume in 1 s (FEV1) in relation to forced vital capacity, as these are used to determine the stage of COPD [47,48]. Moreover, more longitudinal studies are needed to confirm and expand previous results, since only three studies larger than 12 weeks were found, with 6 [37], 12 [42], and 24 [40] months, respectively. In addition, all details of the FITT principle should be described, as well as the exercise (which was not the case in some included studies [22,24,27,29,30,31,34,35,37,38,41]), thereby avoiding the exact replication of the study. Furthermore, future reviews that include additional types of treatments, such as educational programs, will be excluded from the present study, as it has already excluded 11 articles based on this criterion (see Figure 1).

Based on the findings of this scoping review, it is evident that physical exercise interventions, whether focused on cardiorespiratory, strength, or combined modalities, can elicit meaningful improvements in functional capacity and quality of life among individuals with COPD. While not all studies demonstrated uniformly positive outcomes, the heterogeneity in program characteristics, including training intensity, frequency, duration, and exercise modality, likely explains this variability. Importantly, several studies revealed that even low-to-moderate intensities, especially when applied consistently over time, could yield significant clinical benefits, particularly in older adults.

This review also contributes novel insights by including a broader range of training modalities and more precisely defined eligibility criteria than prior systematic reviews [8,9,10,11,12]. The inclusion of home-based, community-based, and high-intensity protocols, alongside traditional supervised programs, enhances the applicability of the findings across different healthcare settings and patient profiles. Taken together, these results reinforce the value of tailored and progressive exercise prescriptions for COPD individuals, emphasizing the need for individualized program design that considers age, baseline functional capacity, and the specific goals of intervention. Future research should focus on long-term adherence, progression strategies, and the integration of multi-domain outcomes to further inform clinical guidelines.

## 5. Conclusions

The present scoping review serves as an additional complement to the general recommendations for exercise prescription, specifically in cardiorespiratory, strength, and combined training.

Through the analysis of the studies, it was possible to conclude that walking exercise can be the only training to improve walking distance, maximal oxygen uptake, tidal volume, decrease heart rate measures at rest or in submaximal intensities, dyspnea, respiratory rate while exercising, and St. George’s Respiratory Questionnaire scores. Moreover, cycling was another valid type of exercise to improve maximal oxygen uptake, resistance in the upper and lower limbs, dyspnea, and quality of life, while decreasing blood pressure values.

In addition, when analyzing strength training studies, it was found that all protocols improved strength. While this is expected, it is worth noting that some of these protocols employed lower intensities than those recommended (e.g., elastic bands with no additional percentage of 1RM). Overall, this type of training contributed to better participant satisfaction with their performance while decreasing perceptions of dyspnea and fatigue. However, no benefits related to specific measures of COPD were found.

Finally, the combined training regimen included continuous aerobic exercise and resistance training for both upper and lower limbs, resulting in improvements in aerobic capacity, as measured by maximal oxygen uptake, dyspnea, exercise tolerance, forced vital capacity, oxygen saturation, and quality of life. Additionally, resting heart rate and blood pressure decreased.

## Figures and Tables

**Table 1:** Full search strategy for each database.

Database	Specificities of the Databases	Search Strategy
PubMed	None to report	((((“exercise”[MeSH Terms] OR “exercise”[All Fields] OR “exercises”[All Fields] OR “exercise therapy”[MeSH Terms] OR (“exercise”[All Fields] AND “therapy”[All Fields]) OR “exercise therapy”[All Fields] OR “exercise s”[All Fields] OR “exercised”[All Fields] OR “exerciser”[All Fields] OR “exercisers”[All Fields] OR “exercising”[All Fields]) AND “prescription*”[All Fields]) OR ((“education”[MeSH Subheading] OR “education”[All Fields] OR “training”[All Fields] OR “education”[MeSH Terms] OR “train”[All Fields] OR “train s”[All Fields] OR “trained”[All Fields] OR “training s”[All Fields] OR “trainings”[All Fields] OR “trains”[All Fields]) AND “program*”[All Fields]) OR ((“exercise”[MeSH Terms] OR “exercise”[All Fields] OR “exercises”[All Fields] OR “exercise therapy”[MeSH Terms] OR (“exercise”[All Fields] AND “therapy”[All Fields]) OR “exercise therapy”[All Fields] OR “exercise s”[All Fields] OR “exercised”[All Fields] OR “exerciser”[All Fields] OR “exercisers”[All Fields] OR “exercising”[All Fields]) AND “periodization*”[All Fields])) AND (“pulmonary disease, chronic obstructive”[MeSH Terms] OR (“pulmonary”[All Fields] AND “disease”[All Fields] AND “chronic”[All Fields] AND “obstructive”[All Fields]) OR “chronic obstructive pulmonary disease”[All Fields] OR (“chronic”[All Fields] AND “obstructive”[All Fields] AND “pulmonary”[All Fields] AND “disease”[All Fields]) OR “copd*”[All Fields]))
Web of Science	None to report	((((ALL=(exercise prescription*)) OR ALL=(training program*)) OR ALL=(exercise periodization*)) AND ALL=(chronic obstructive pulmonary disease )) OR ALL=(COPD*)
Scopus	Search for title and abstract also includes keywords	(TITLE-ABS-KEY (exercise AND prescription*) OR TITLE-ABS-KEY training AND program*) OR TITLE-ABS-KEY (exercise AND periodization*) AND TITLE-ABS-KEY (chronic AND obstructive AND pulmonary AND disease) OR TITLE-ABS-KEY (copd*)
SPORTDiscus	Search for title only	((exercise prescription*OR training program* OR exercise periodization*) AND (chronic obstructive pulmonary disease OR COPD*)).

## Data Availability

Not applicable.

## Supplementary Materials

The following supporting information can be downloaded at: https://www.mdpi.com/article/10.3390/medicina61060970/s1, File S1: PRISMA 2020 Checklist; File S2: Criteria for assessing study quality and bias.
